# Complete genome sequence of *Dehalobacter* sp. strain CP, an organohalide-respiring anaerobe from a chlorophenol-dechlorinating consortium

**DOI:** 10.1128/mra.00092-25

**Published:** 2025-06-10

**Authors:** Xiuying Li, Jingjing Wang, Huijuan Jin, Yiru Cui, Hao Zhong, Tongyue Zhou, Jun Yan

**Affiliations:** 1 Key Laboratory of Pollution Ecology and Environmental Engineering, Institute of Applied Ecology, Chinese Academy of Sciences 74763 , Shenyang, Liaoning, China; 2 University of Chinese Academy of Sciences 74763 , Beijing, China; University of Maryland School of Medicine, Baltimore, Maryland, USA

**Keywords:** organohalide respiration, reductive dechlorination, *Dehalobacter*, chlorophenol, groundwater bioremediation

## Abstract

Strain CP, a novel member affiliated with the genus *Dehalobacter*, was identified in an anaerobic consortium capable of 4-chlorophenol-to-phenol reductive dechlorination. The complete genome of strain CP comprises a 2.94 Mb circular chromosome with a G + C content of 44.5% and encodes 40 reductive dehalogenase homologous genes.

## ANNOUNCEMENT

An anaerobic consortium capable of reductively dechlorinating 4-chlorophenol (4-CP) to phenol was established in 2018 using a sediment sample collected from the Xi River (Shenyang, Liaoning, China; 41°39′46″N, 123°6′20″E). The 4-CP-dechlorinating consortium was cultivated in basal mineral salt medium (1) supplemented with 5 mM acetate as carbon source, 413 µmol hydrogen as electron donor, and 80 µM 4 CP as electron acceptor. Amplicon sequencing of 16S rRNA gene targeting the prokaryotic V3-V4 region was conducted using published primers 5′-CCTACGGRRBGCASCAGKVRVGAAT-3′ and 5′-GGACTACNVGGGTWTCTAATCC-3′ (2). The enrichment of a *Dehalobacter* population, designated as strain CP, was revealed and likely accounted for the observed 4-CP-dechlorinating activity.

Biomass was collected from 1.6 L of the 4-CP-dechlorinating consortium, and genomic DNA was extracted using the E.Z.N.A. stool DNA Kit (Omega Bio-tek, Norcross, GA) according to the manufacturer’s instructions. Sequencing was performed using a hybrid approach that combined Illumina NovaSeq 6000 platform (Illumina Inc., San Diego, CA) with PacBio Sequel II platform (Pacific Biosciences, Menlo Park, CA). For Illumina sequencing, the library was constructed using the Nextera XT DNA Library Preparation kit (Illumina Advantage, USA), yielding a total of 230,941,488 paired-end reads (2 × 150 bp) with 97.85% of bases achieving Q30 quality. For PacBio sequencing, 5 µg DNA was used to construct a SMRTbell library using the SMRTbell Prep Kit (Pacific Biosciences) according to the manufacturer’s protocol (3). DNA was sheared using a g-TUBE, followed by size selection to obtain fragments larger than 3 kb. The HiFi reads were generated with the CCS module (-min-passes = 3, and -min-rq = 0.99) using SMRT Link v11.0 (Pacific Biosciences). The sequencing yielded a total of 2,943,357 long reads (*N_50_* = 24,279 bp). Raw sequence reads were quality-trimmed using Trimmomatic to remove adaptor contaminants and low-quality reads (4). MetaFlye version 2.9.6 (https://github.com/mikolmogorov/Flye) was used to assemble the sequence reads, identify circular structures, and trim overlapping regions. The replication origin in the circular genome was rotated to a position upstream of the *dnaA* gene using Circlator (5). Genome annotation was performed using the NCBI Prokaryotic Genome Annotation Pipeline v6.7 (6).

The metagenome-assembled strain CP genome is represented by a single contig comprising a 2,935,772 bp circular chromosome with a G + C content of 44.5%. A total of 2,831 coding sequences, 53 tRNAs, and 12 rRNAs, including four copies each of the 5S, 16S, and 23S rRNA genes, were annotated. BLASTn analysis indicated that strain CP’s 16S rRNA gene is closely related to that of *Dehalobacter* sp. strain TCP1 (GenBank accession number JX999723) with 99.7% sequence similarity, supporting the taxonomic placement of strain CP into the genus *Dehalobacter*. Strain CP genome contains 40 non-identical reductive dehalogenase (*rdh*) subunit A genes, of which 13 are adjacent to a downstream *rdhB* gene coding for a membrane anchor protein. The genome also features several gene clusters relevant to the respiratory chain including those encoding a hydrogenase-4 complex (locus tag # AAH944_07925), four nickel-dependent hydrogenase complexes (locus tags # AAH944_03070, AAH944_02445, AAH944_04680, AAH944_07900), three NADH-dependent [FeFe] hydrogenase complexes (locus tags # AAH944_03450, AAH944_08330, AAH944_03180).

## Data Availability

The sequences of strain CP have been deposited in GenBank under the accession numbers MW380252 (16S rRNA gene) and CP157399 (Genome sequence). The BioProject and BioSample accession numbers are PRJNA1105332 and SAMN41100050, respectively. The raw sequencing reads have been deposited in the SRA under the accession numbers SRR32041004 (PacBio) and SRR32041005 (Illumina).
